# Supplementary material for: Phenotype loss is associated with widespread divergence of the gene regulatory landscape in evolution
Source: Nat Commun. 2018 Nov 9;9:4737. doi: 10.1038/s41467-018-07122-z (PMC6226452; doi:10.1038/s41467-018-07122-z)
Supplement: Supplementary file 1 — Supplementary Information [file 41467_2018_7122_MOESM1_ESM.pdf]

Supplementary Information for

Phenotype loss is associated with widespread divergence of the gene regulatory landscape in evolution

Juliana G. Roscito[1,2,3,#], Katrin Sameith[1,2,3,#], Genis Parra[1,2,3,#], Bjoern E. Langer[1,2,3], Andreas Petzold[4], Claudia Moebius[1], Marc Bickle[1], Miguel Trefaut Rodrigues[5], and Michael Hiller[1,2,3*]

[1]Max Planck Institute of Molecular Cell Biology and Genetics, Dresden, Germany
[2]Max Planck Institute for the Physics of Complex Systems, Dresden, Germany
[3]Center for Systems Biology Dresden, Germany
[4]Center for Regenerative Therapies TU Dresden, Germany
[5]Instituto de Biociências, Universidade de São Paulo, Brazil

[#] Joint first authors

* To whom correspondence should be addressed:
Michael Hiller
Computational Biology and Evolutionary Genomics, Max Planck Institute of Molecular Cell Biology and Genetics & Max Planck Institute for the Physics of Complex Systems, Dresden, Germany.
Tel: +49 351 210 2781
Fax: +49 351 210 1209
Email: hiller@mpi-cbg.de

The Supplementary material contains Supplementary Figures 1-17

Supplementary Tables 1-23 are provided as sheets in a separate Excel file.

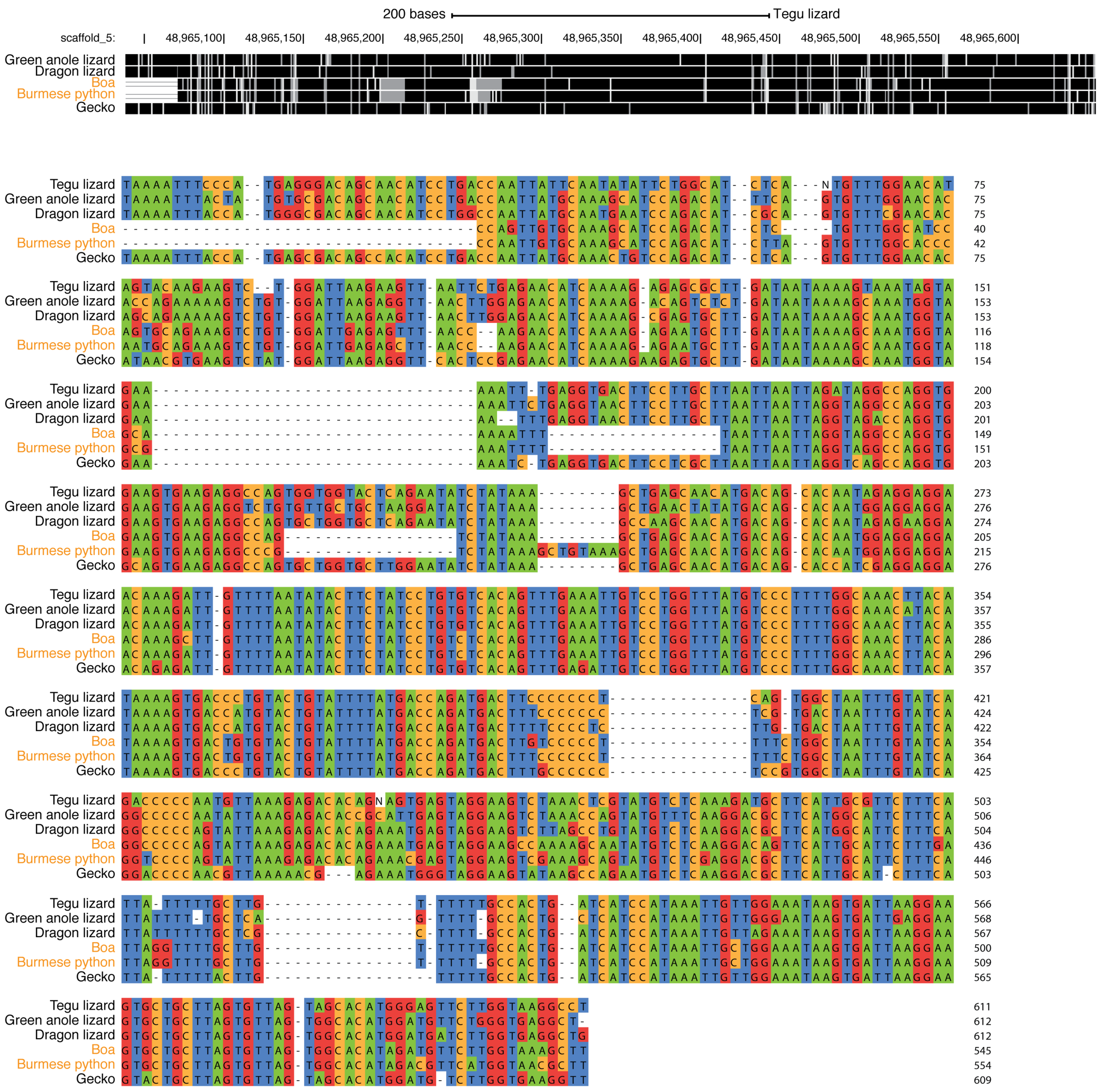

Supplementary Figure 1: Genome Browser screenshot and sequence alignment of the CNE overlapping the 5’ end of the known *Shh* ZRS limb enhancer.
top: Multiple sequence alignment visualization in the UCSC genome browser. Sequences are visualized on a grey-scale; the darker the color, the higher is the sequence similarity in the alignment. Double horizontal lines indicate sequence that does not align between the reference (tegu lizard) and the query species.
bottom: Detailed sequence alignment. For space considerations, only the sequences of Episquamata species are shown. Columns with gaps in all episquamates have aligning sequence in other species that are not shown.

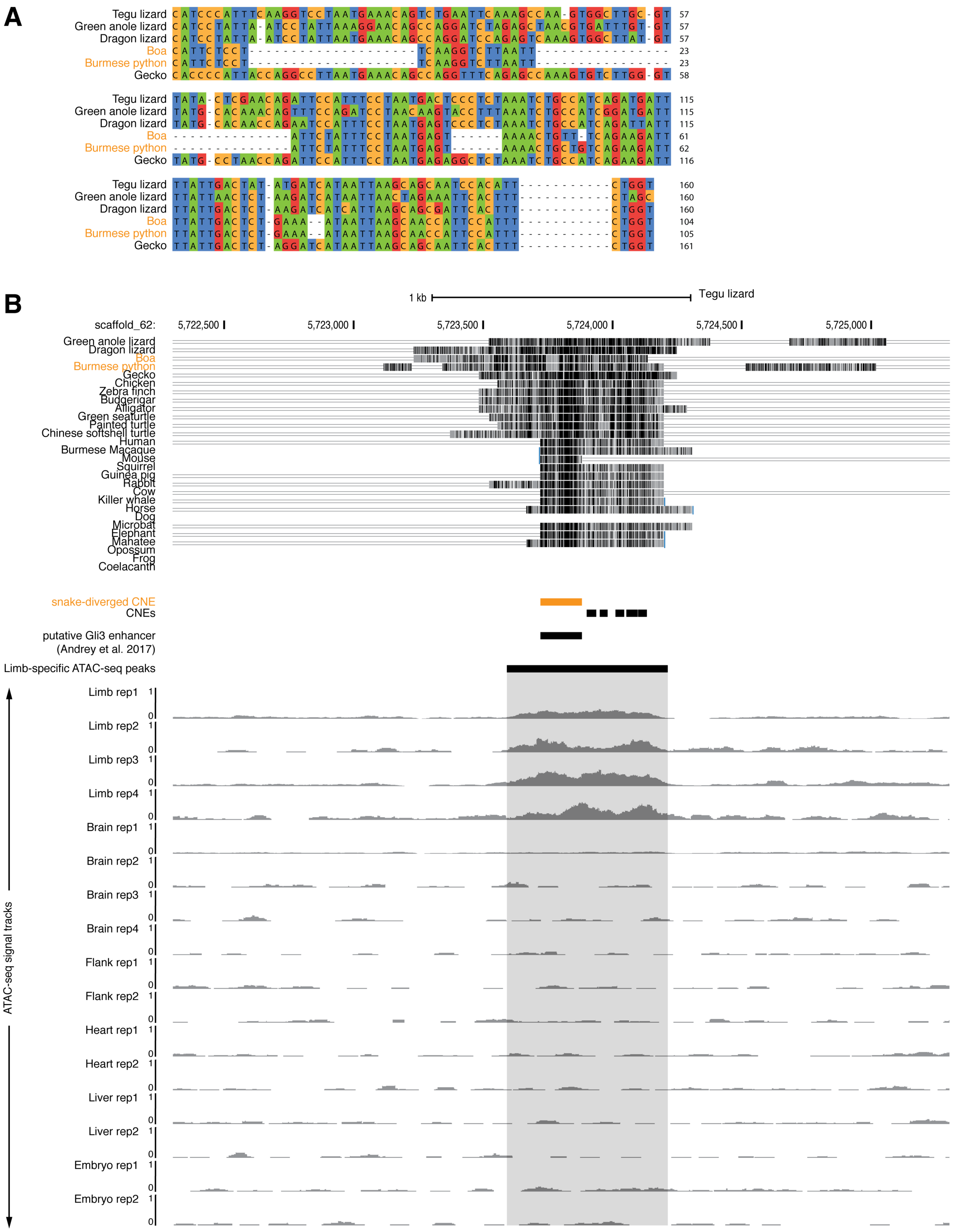

Supplementary Figure 2: Example of a snake-diverged CNE regulating the limb-related transcription factor *Gli3*.

(A) Alignment of this CNE shows several substitutions and small deletions in the snake lineage. Only the sequences of Episquamata species are shown. Columns with gaps in all episquamates have aligning sequence in other species that are not shown.

(B) UCSC genome browser screenshot of a larger genomic region shows that this snake-diverged CNE (orange) is conserved among Amniota species (multiple alignment visualization as in Supplementary Figure 1). Our ATAC-seq signal tracks show that the snake-diverged CNE overlaps a genomic region with accessible chromatin in limb but not in other developing tissues of the tegu lizard. Furthermore, this CNE overlaps a *Gli3* enhancer identified by Capture-C [1].

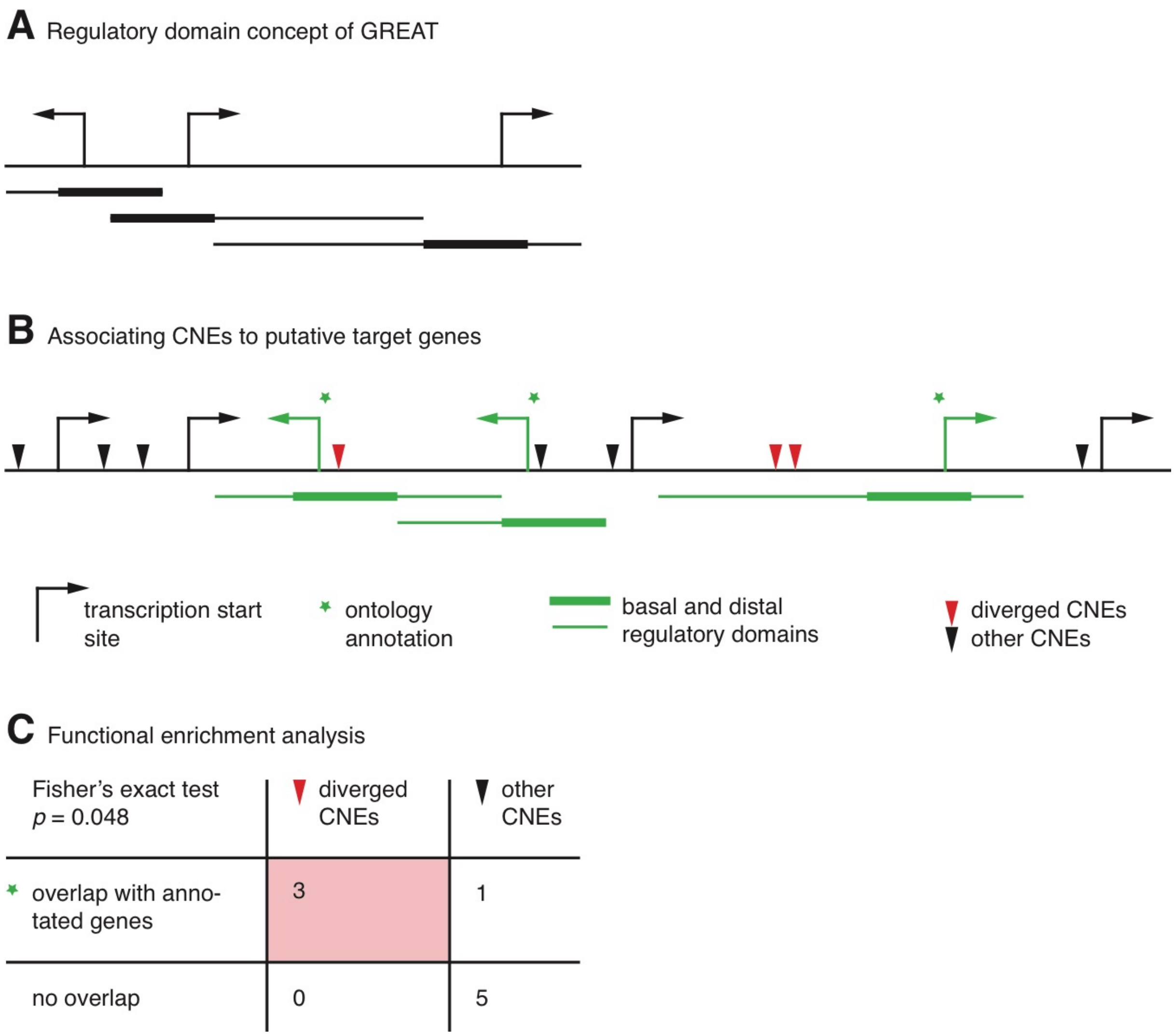

Supplementary Figure 3: Regulatory domain definition and statistical test to assess if diverged CNEs are significantly associated with genes annotated with particular functions.

(A) GREAT regulatory domain concept (adapted from the GREAT website). Similar to reference [2], we define regulatory domains for each gene as a basal (promoter-associated) domain of 5 Kb upstream and 1 Kb downstream of the transcription start site and a distal domain extending up to the basal domain of the next gene or at most 300 Kb in either direction.

(B) To determine putative target genes for each CNE, we ask which regulatory domains overlap this CNE. Genes annotated with a particular function (foreground) are in green, all others are in black (background).

(C) A one-sided Fisher's exact test is applied to test whether diverged CNEs exhibit a significantly higher overlap with regulatory domains of genes annotated with a particular function.

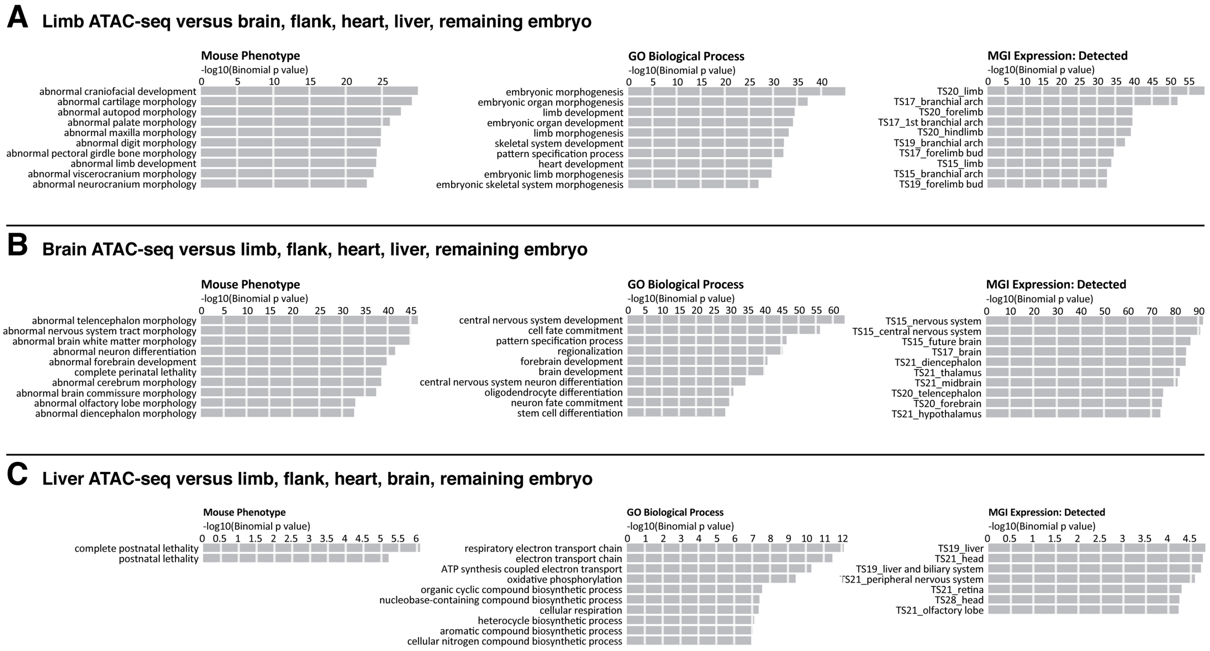

Supplementary Figure 4: GREAT enrichments of ATAC-seq peaks from embryonic tissues of the tegu lizard.

Tegu ATAC-seq peaks that show differential signal in one tissue versus all other examined tissues were mapped to the mouse mm10 genome using liftOver [3] (parameters '*-minMatch=0.1*') and subjected to a GREAT analysis [2] using default parameters. Up to 10 top enriched terms per ontology are shown. These enrichments reveal knockout phenotypes, gene expression patterns and functional annotations that are expected for regulatory elements active in limb, brain and liver tissue. No enrichments were found for embryo, flank, and heart ATAC-seq peaks due to very low numbers of tissue-specific peaks.

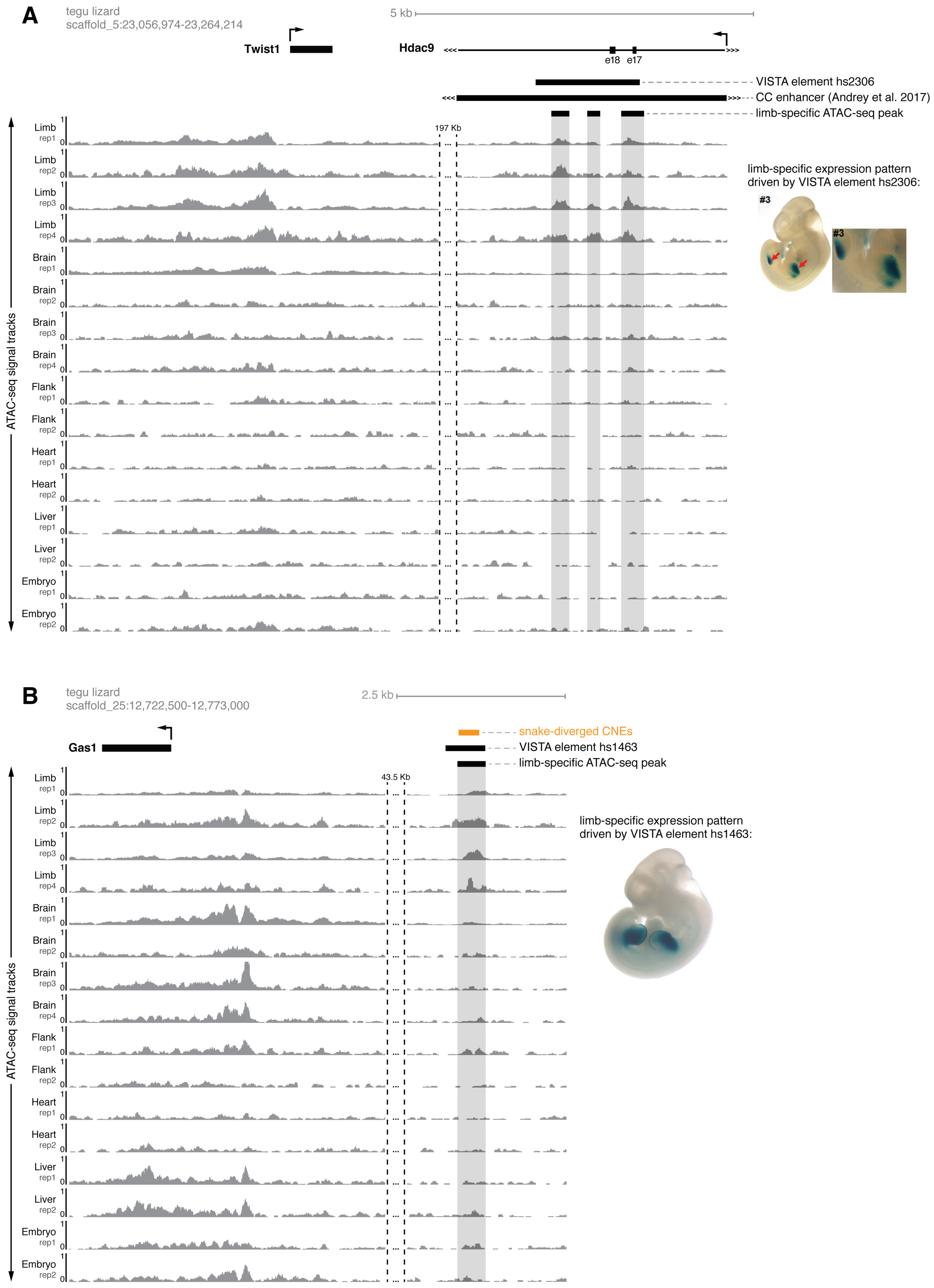

Supplementary Figure 5: Limb-specific ATAC-seq peaks and snake-diverged CNEs overlap functionally validated limb regulatory elements.

(A) Limb-specific ATAC-seq peaks overlap a known *Twist1* enhancer that contacts the *Twist1* promoter [1,4]. This region also overlaps an experimentally characterized VISTA enhancer element hs2306 [5] that drives expression only in the limbs at mouse embryonic day E11.5 (image taken from https://enhancer.lbl.gov/cgi-bin/imagedb3.pl?form=presentation&show=1&experiment_id=2306&organism_id=1).
(B) A snake-diverged CNE marked by a limb-specific ATAC-seq peak located upstream of *Gas1*, a positive regulator of *Shh* activity [6], overlaps an experimentally tested VISTA enhancer element hs1463 [5] that drives expression only in limbs (image taken from https://enhancer.lbl.gov/cgi-bin/imagedb3.pl?form=presentation&show=1&experiment_id=1463&organism_id=1).

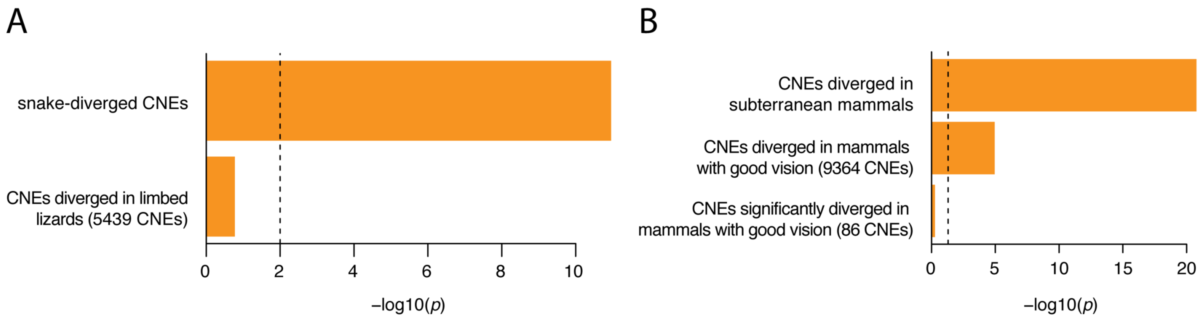

Supplementary Figure 6: Limb (A) and eye (B) regulatory elements overlap CNEs diverged in snakes and CNEs diverged in subterranean mammals significantly more often than regulatory elements active in non-limb or non-eye tissues.
Limb regulatory elements have no significant overlap with the top 5,439 CNEs diverged in limbed lizards that was used as a control set (A). While eye regulatory elements also have a significant overlap with the control set comprising the top 9,364 CNEs diverged in mammals with good vision (most of these having a FDR value greater than our cutoff of 0.005), the significance is substantially lower compared to the CNEs significantly diverged in subterranean mammals (FDR < 0.005). Please note that we are investigating here whether regulatory elements active in limb or eye tissue overlap diverged CNEs significantly more often than regulatory elements active in non-limb or non-eye tissues, which is different from asking whether diverged CNEs overlap certain regulatory data sets significantly more often than non-diverged CNEs (Figure 2 and 3, main text). We combined all limb/eye regulatory elements for this analysis. Bars depict *p*-values derived by a one-sided Fisher’s exact test.

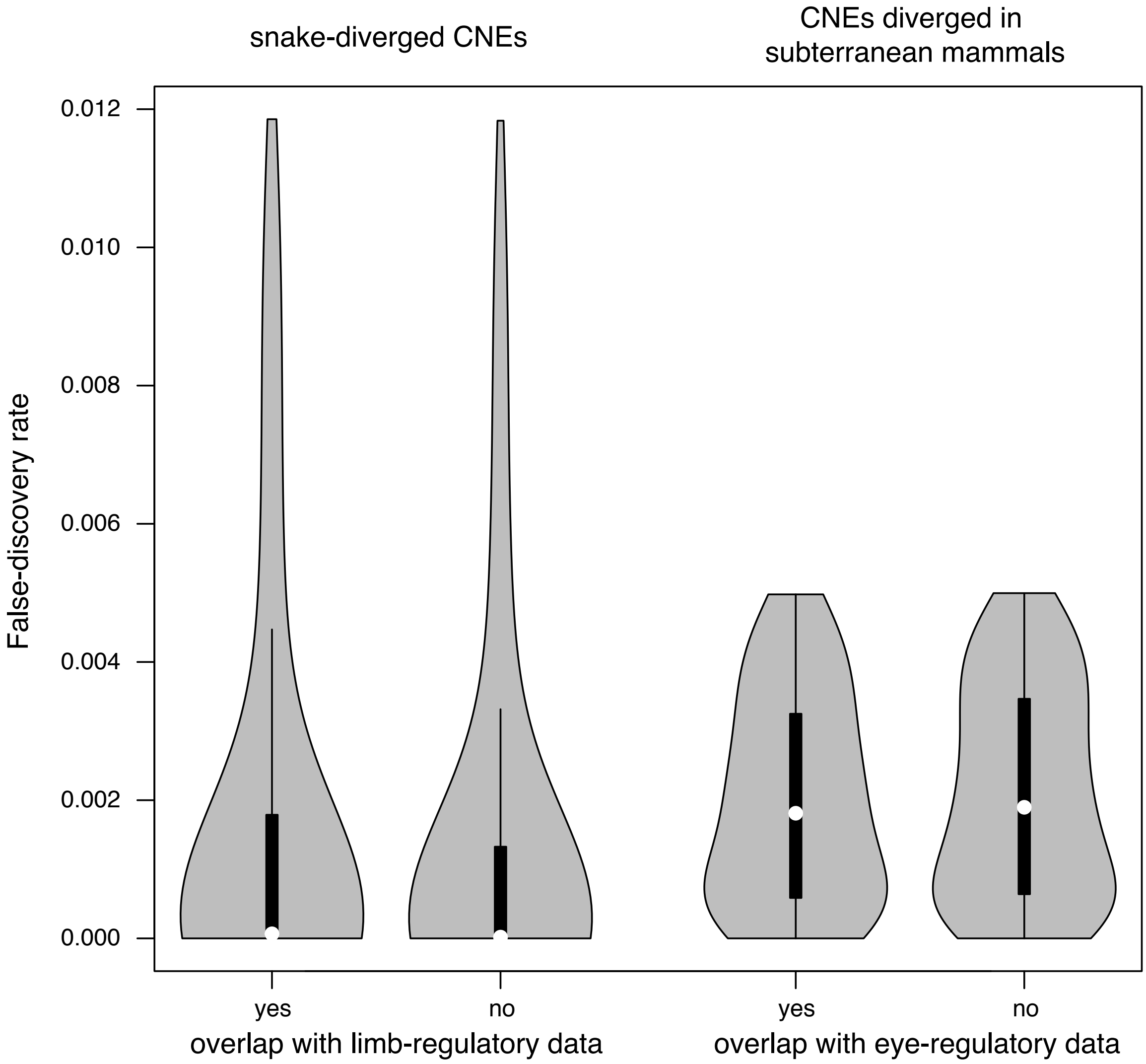

Supplementary Figure 7: FDR distribution between diverged CNEs that do and do not overlap regulatory data.
The distribution of FDR values is similar between the 933 snake-diverged CNEs that overlap limb-regulatory data and the remaining snake-diverged CNEs that do not (left side). Likewise, the distribution of FDR values is similar between the 575 CNEs diverged in subterranean mammals that overlap eye-regulatory data and the remaining diverged CNEs that do not (right side). Violin plots are used to visualize the FDR value distributions (white dot represents the median, thick black lines represent the interquartile range, and thin black lines represent the 95% confidence interval).

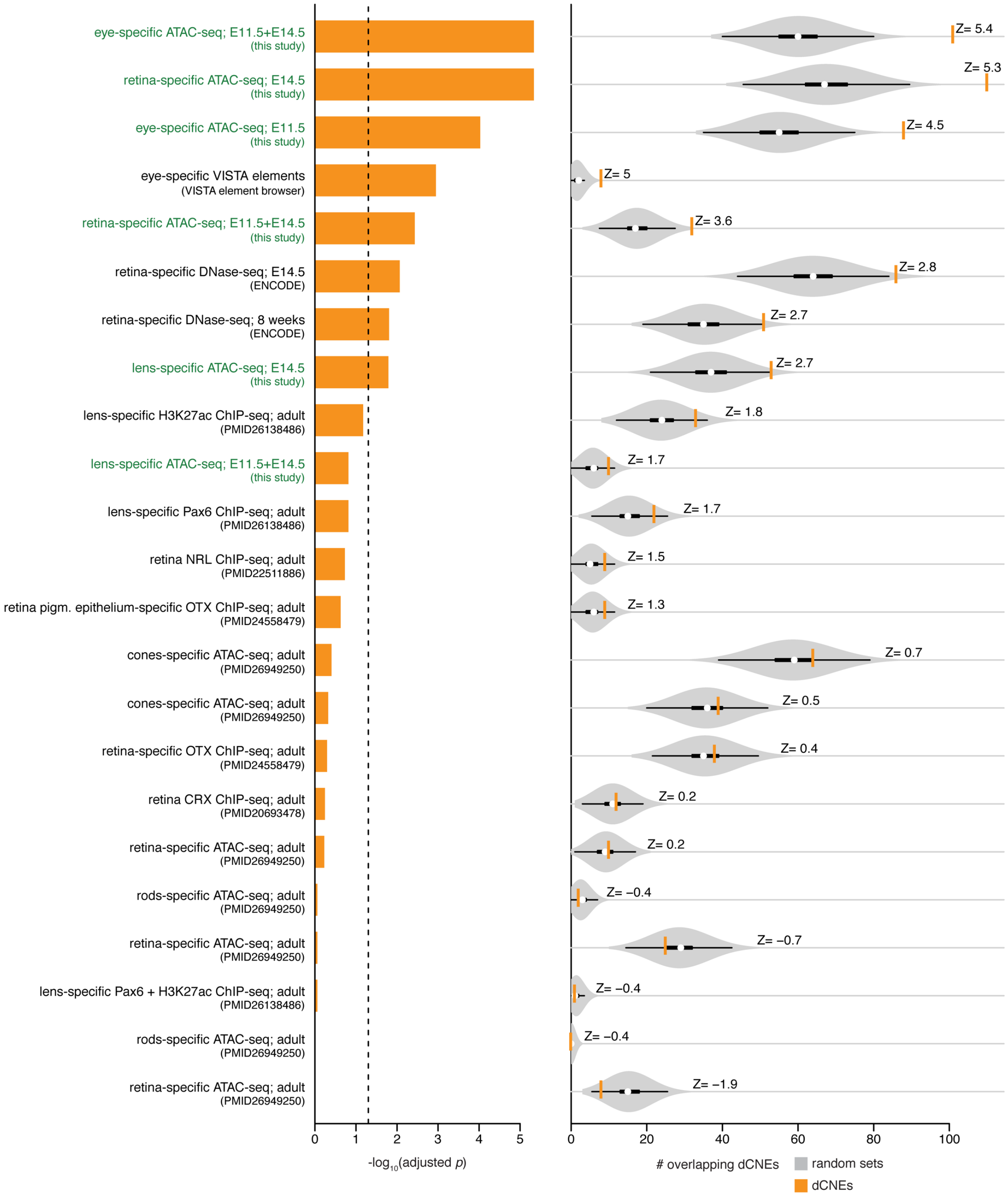

Supplementary Figure 8: Snake-diverged CNEs significantly overlap eye regulatory elements active in whole eye, retina, and lens.
Left: orange bars represent the Benjamini & Hochberg adjusted *p*-values derived by a one-sided Fisher's exact test and show the significance of the overlap between snake-diverged CNEs and eye regulatory datasets derived from functional experiments in multiple mouse embryonic and adult eye tissues. ATAC-seq datasets generated in this study are highlighted in green. Right: Observed (orange vertical bar) and expected number (grey violin plots, based on 10,000 random subsets sampled from all CNEs)

of snake-diverged CNEs overlapping eye regulatory elements. The thick box inside the violin plot indicates the first quartile, the median, and the third quartile. The Z-score measuring the number of standard deviations that the observed number is above the random expectation is indicated.

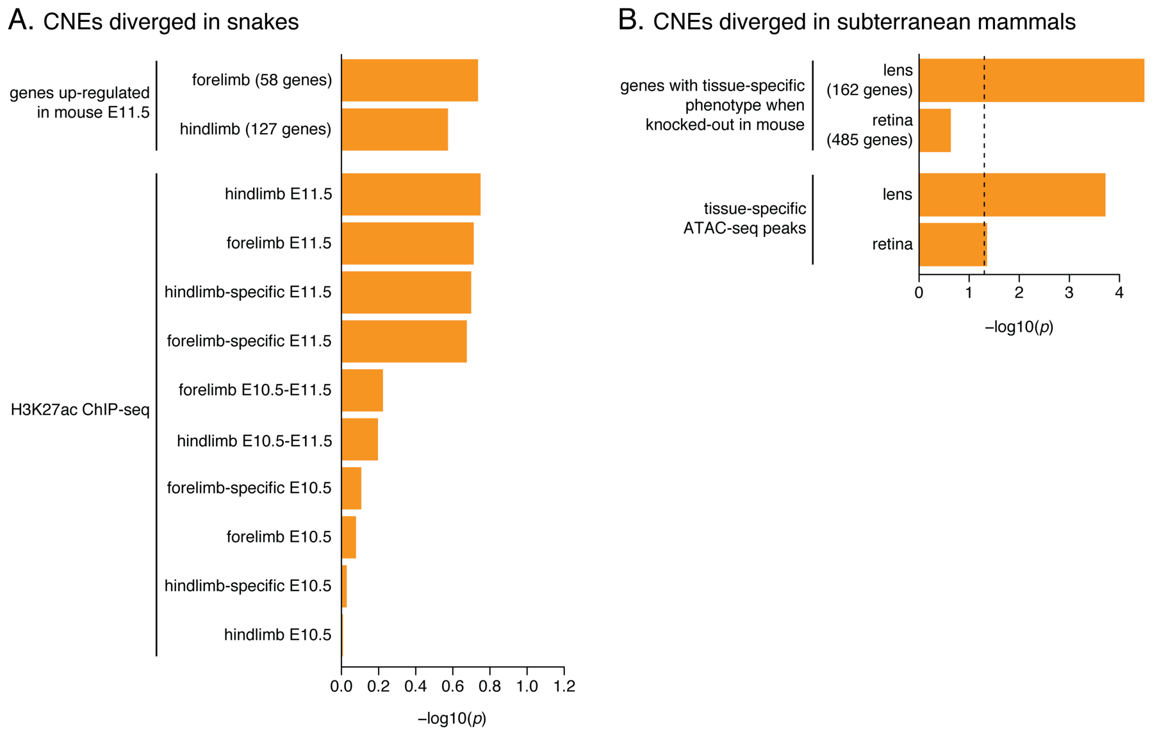

Supplementary Figure 9: Overlap of diverged CNEs with genes and regulatory elements that are specifically active in particular tissues within limbs or within eyes.
(A) Snake-diverged CNEs are not preferentially associated with fore- or hindlimb up-regulated genes (test set comprises 58 and 127 genes up-regulated in mouse E11.5 fore- and hindlimbs, respectively [7]), or with fore- or hindlimb-specific enhancers (test set of fore- and hindlimb-specific enhancers determined with H3K27ac ChIP-seq in mouse E10.5 and E11.5 limb buds[7]). The lack of enrichments for any of the tested sets is most likely explained by the low number of elements in each test set.
(B) CNEs diverged in subterranean mammals are preferentially associated with genes which result in lens-specific phenotypes when knocked-out in mouse, compared to genes which result in retina-specific phenotypes (test set of 162 and 485 lens and retina genes, respectively; obtained from Mouse Genome Informatics http://www.informatics.jax.org/). These CNEs also overlap lens-specific regulatory elements significantly more often than retina-specific regulatory elements (mouse ATAC-seq data from this study).
Bars depict *p*-values derived by a one-sided Fisher's exact test.

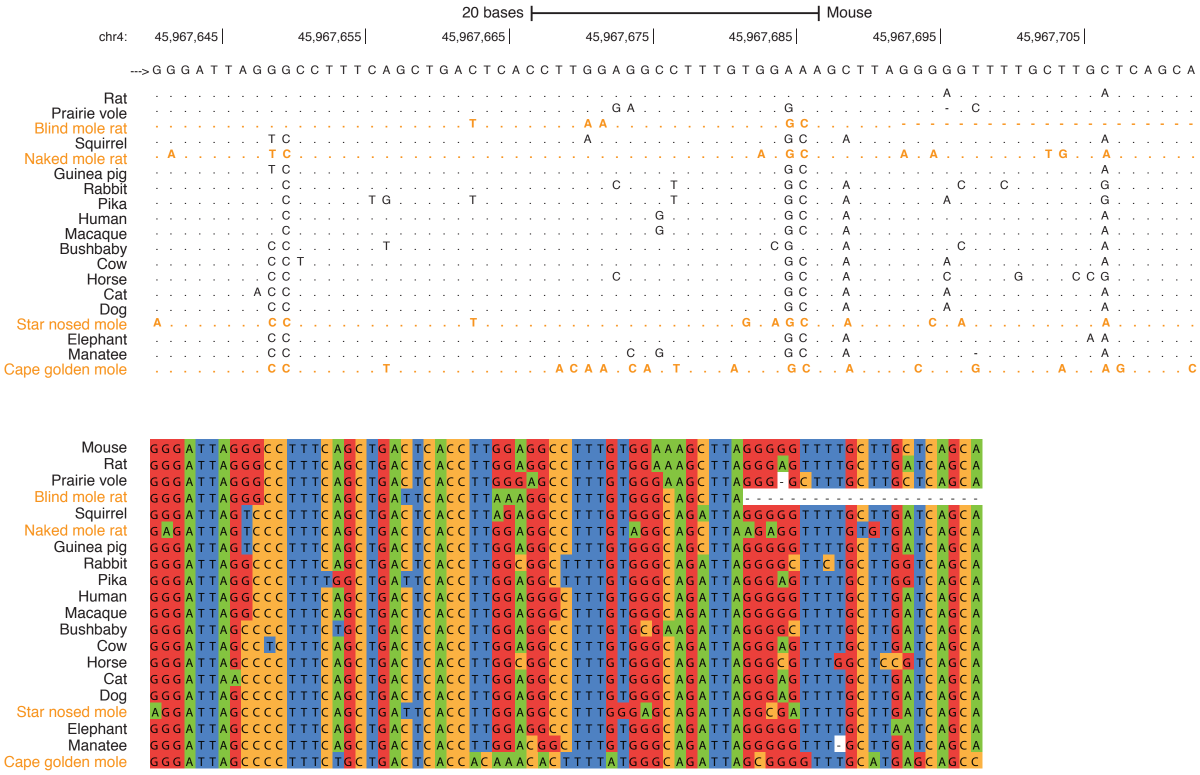

Supplementary Figure 10: Sequence alignment of the CNE shown in Figure 4D. This CNE exhibits higher sequence divergence in subterranean mammals, as shown in both alignment visualizations. In addition to an excess of substitutions, the blind mole rat has a deletion at the 3' end of this CNE. The CNE is 20 Kb upstream of the RNA granule component *Tdrd7* that plays an important role in lens development [8].

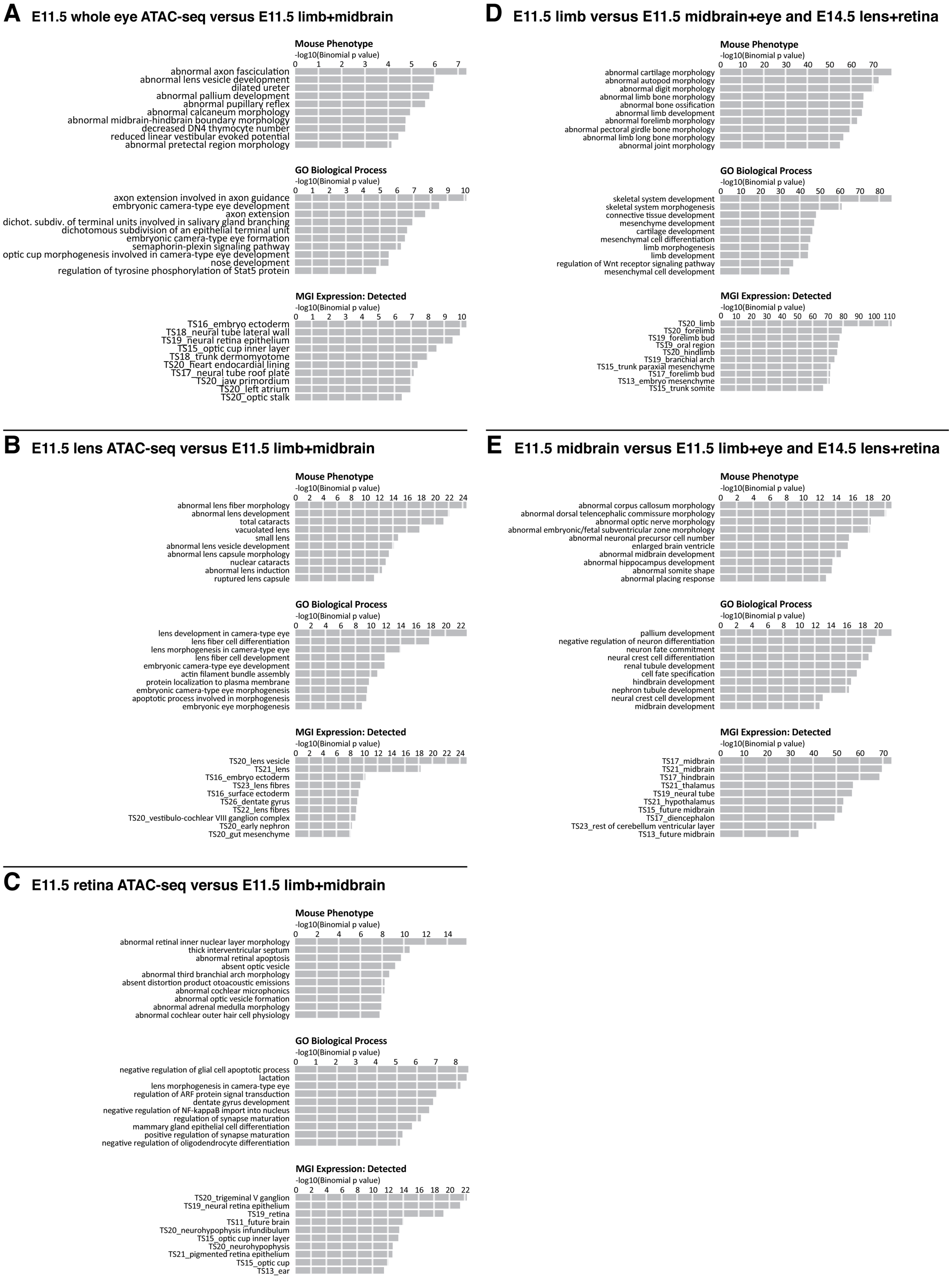

Supplementary Figure 11: GREAT enrichments of mouse eye-, lens-, retina-, limb- and midbrain-specific ATAC-seq peaks.

These enrichments reveal knockout phenotypes, gene expression patterns and functional annotations that are expected for regulatory elements active in these respective tissues. Up to 10 top enriched terms per ontology are shown.

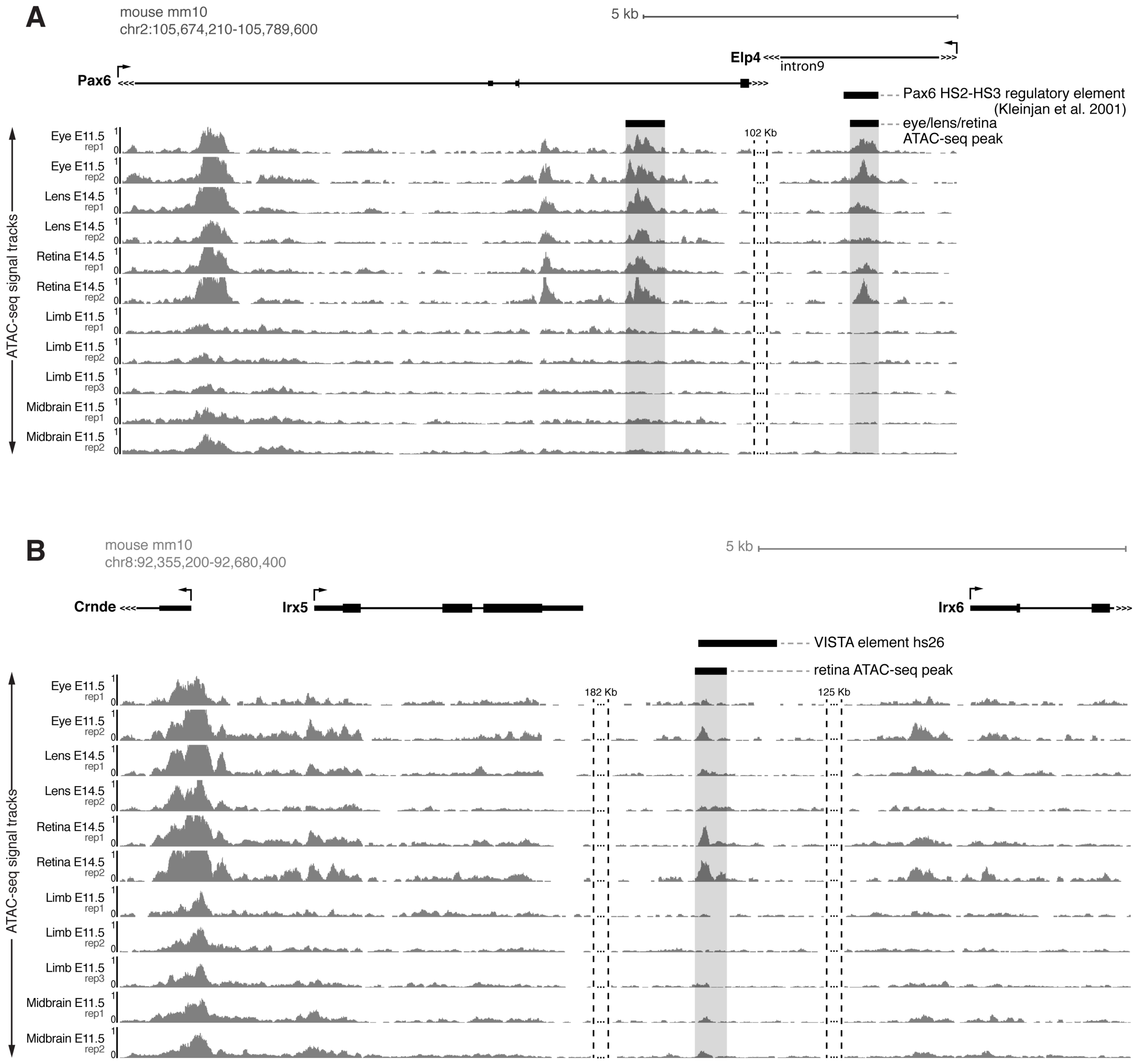

Supplementary Figure 12: Eye-specific ATAC-seq peaks overlap functionally validated eye regulatory elements.

(A) ATAC-seq peaks in eye, lens and retina tissues overlap an experimentally characterized *Pax6* regulatory element [9].

(B) A retina-specific ATAC-seq peak located in between *Irx5* and *Irx6* overlaps the VISTA enhancer element hs26 [5] that drives expression in eyes and other tissues.

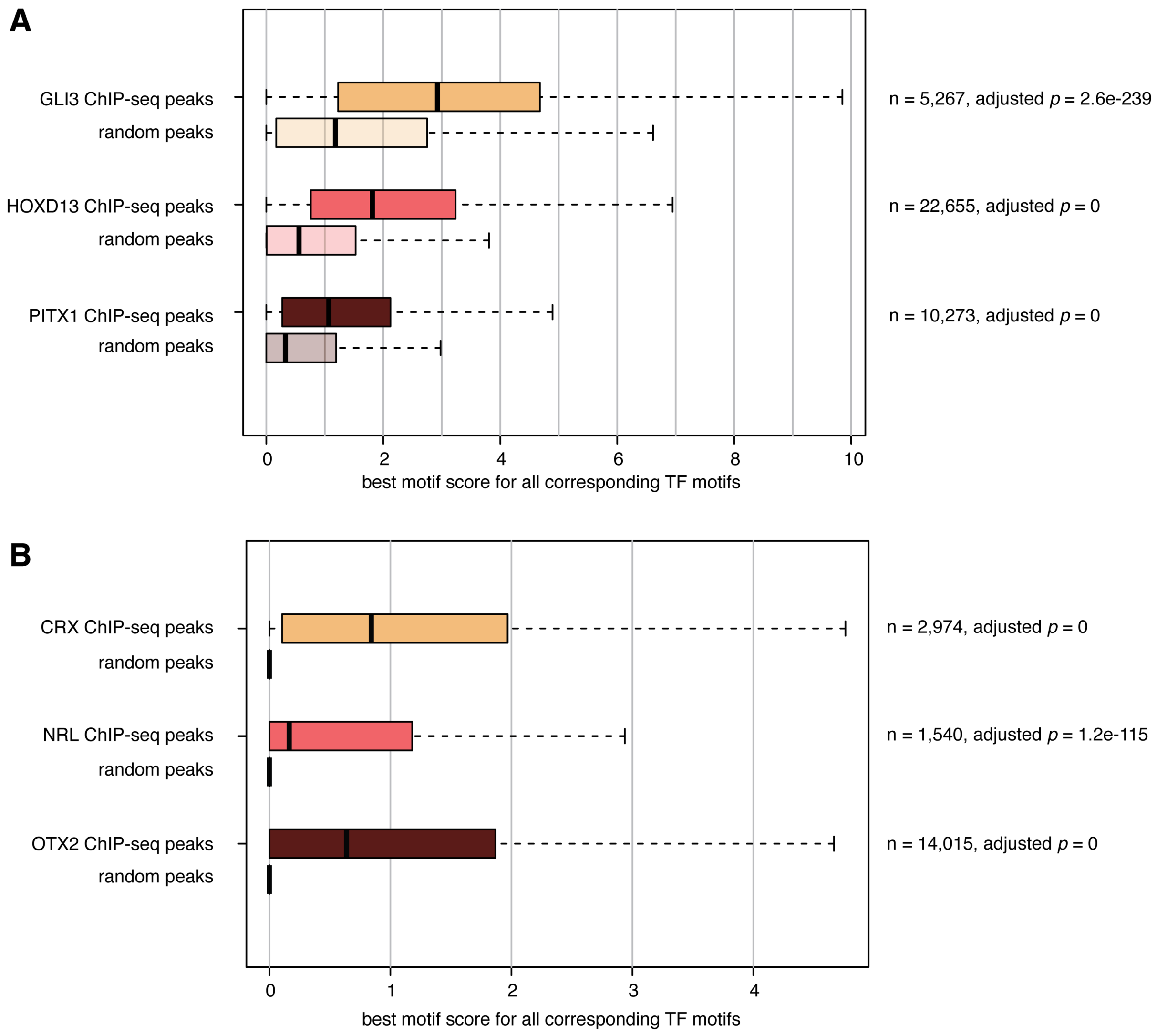

Supplementary Figure 13: Transcription factor (TF) motif scores for mouse genomic regions bound by the TF.

(A) Mouse genomic regions that are bound by GLI3 [10], HOXD13 [11] and PITX1 [12], according to ChIP-seq experiments in mouse limb tissue, have significantly higher motif scores for these TFs, compared to the same number of randomly selected genomic regions of the same size.

(B) Mouse genomic regions that are bound by CRX [13], NRL [14] and OTX2 [15], according to ChIP-seq experiments in mouse eye tissue, have significantly higher TF motif scores for these TFs, compared to the same number of randomly selected genomic regions of the same size. The ChIP-seq peaks for the three eye transcription factors are smaller than the peaks in (A) (average 223 vs. 1140 bp), explaining why most of the randomly selected controls have a score of 0.

Benjamini & Hochberg adjusted *p*-values derived by a one-sided Wilcoxon rank sum test.

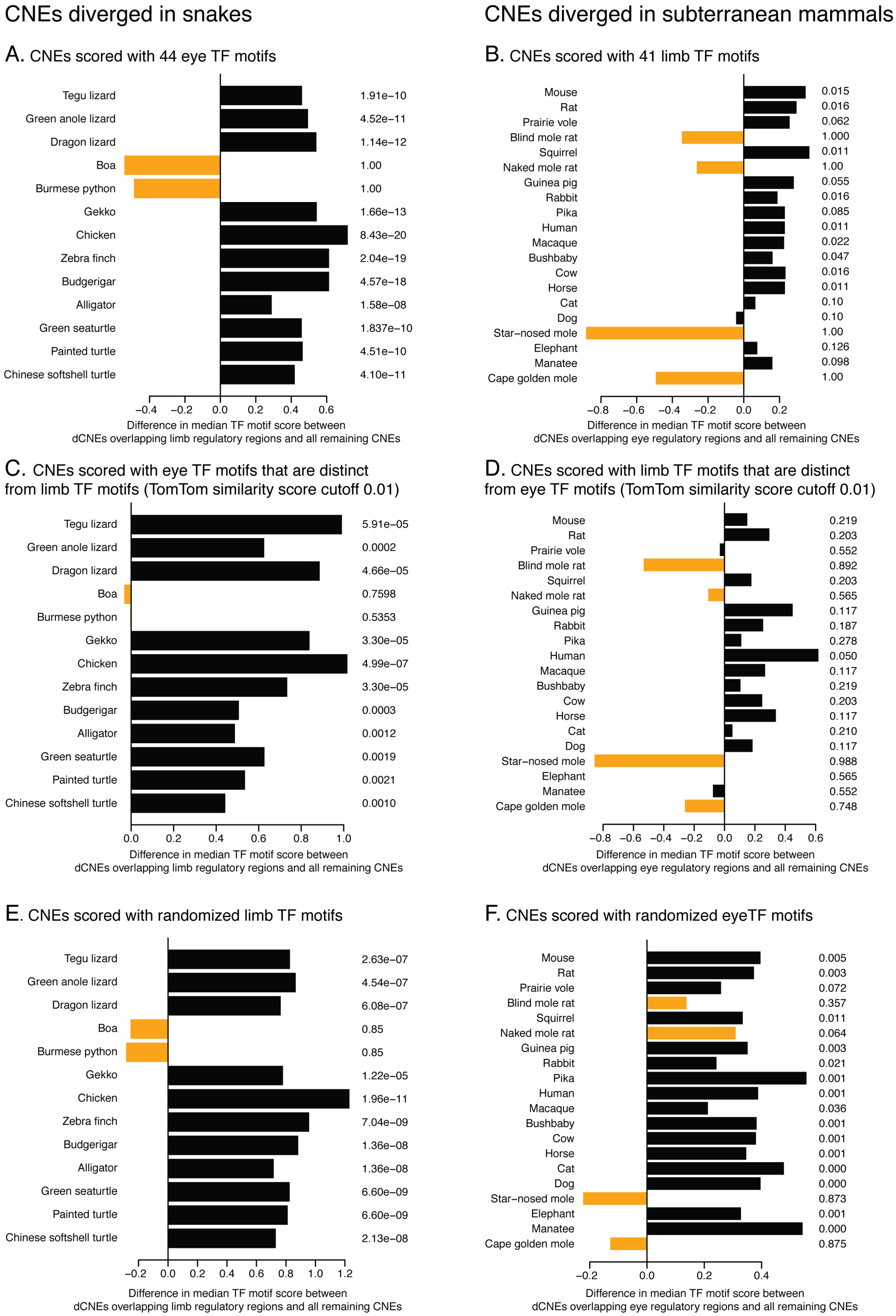
CNEs diverged in snakes
A. CNEs scored with 44 eye TF motifs
Tegu lizard 1.91e−10
Green anole lizard 4.52e−11
Dragon lizard 1.14e−12
Boa 1.00
Burmese python 1.00
Gekko 1.66e−13
Chicken 8.43e−20
Zebra finch 2.04e−19
Budgerigar 4.57e−18
Alligator 1.58e−08
Green seaturtle 1.837e−10
Painted turtle 4.51e−10
Chinese softshell turtle 4.10e−11
−0.4 −0.2 0.0 0.2 0.4 0.6
Difference in median TF motif score between
dCNEs overlapping limb regulatory regions and all remaining CNEs
CNEs diverged in subterranean mammals
B. CNEs scored with 41 limb TF motifs
Mouse 0.015
Rat 0.016
Prairie vole 0.062
Blind mole rat 1.000
Squirrel 0.011
Naked mole rat 1.00
Guinea pig 0.055
Rabbit 0.016
Pika 0.085
Human 0.011
Macaque 0.022
Bushbaby 0.047
Cow 0.016
Horse 0.011
Cat 0.10
Dog 0.10
Star−nosed mole 1.00
Elephant 0.126
Manatee 0.098
Cape golden mole 1.00
−0.8 −0.6 −0.4 −0.2 0.0 0.2
Difference in median TF motif score between
dCNEs overlapping eye regulatory regions and all remaining CNEs
C. CNEs scored with eye TF motifs that are distinct from limb TF motifs (TomTom similarity score cutoff 0.01)
Tegu lizard 5.91e−05
Green anole lizard 0.0002
Dragon lizard 4.66e−05
Boa 0.7598
Burmese python 0.5353
Gekko 3.30e−05
Chicken 4.99e−07
Zebra finch 3.30e−05
Budgerigar 0.0003
Alligator 0.0012
Green seaturtle 0.0019
Painted turtle 0.0021
Chinese softshell turtle 0.0010
0.0 0.2 0.4 0.6 0.8 1.0
Difference in median TF motif score between
dCNEs overlapping limb regulatory regions and all remaining CNEs
D. CNEs scored with limb TF motifs that are distinct from eye TF motifs (TomTom similarity score cutoff 0.01)
Mouse 0.219
Rat 0.203
Prairie vole 0.552
Blind mole rat 0.892
Squirrel 0.203
Naked mole rat 0.565
Guinea pig 0.117
Rabbit 0.187
Pika 0.278
Human 0.050
Macaque 0.117
Bushbaby 0.219
Cow 0.203
Horse 0.117
Cat 0.210
Dog 0.117
Star−nosed mole 0.988
Elephant 0.565
Manatee 0.552
Cape golden mole 0.748
−0.8 −0.6 −0.4 −0.2 0.0 0.2 0.4 0.6
Difference in median TF motif score between
dCNEs overlapping eye regulatory regions and all remaining CNEs
E. CNEs scored with randomized limb TF motifs
Tegu lizard 2.63e−07
Green anole lizard 4.54e−07
Dragon lizard 6.08e−07
Boa 0.85
Burmese python 0.85
Gekko 1.22e−05
Chicken 1.96e−11
Zebra finch 7.04e−09
Budgerigar 1.36e−08
Alligator 1.36e−08
Green seaturtle 6.60e−09
Painted turtle 6.60e−09
Chinese softshell turtle 2.13e−08
−0.2 0.0 0.2 0.4 0.6 0.8 1.0 1.2
Difference in median TF motif score between
dCNEs overlapping limb regulatory regions and all remaining CNEs
F. CNEs scored with randomized eyeTF motifs
Mouse 0.005
Rat 0.003
Prairie vole 0.072
Blind mole rat 0.357
Squirrel 0.011
Naked mole rat 0.064
Guinea pig 0.003
Rabbit 0.021
Pika 0.001
Human 0.001
Macaque 0.036
Bushbaby 0.001
Cow 0.001
Horse 0.001
Cat 0.000
Dog 0.000
Star−nosed mole 0.873
Elephant 0.001
Manatee 0.000
Cape golden mole 0.875
−0.2 0.0 0.2 0.4
Difference in median TF motif score between
dCNEs overlapping eye regulatory regions and all remaining CNEs

Supplementary Figure 14: CNE divergence in snakes and in subterranean mammals results in an overall decay of transcription factor binding sites.

(A,C,E) The 933 snake-diverged CNEs that overlap limb regulatory data were scored with (A) 44 eye TF motifs present solely in the eye TF list (Supplementary Tables 20 and 21), (C) motifs of only those eye TFs whose binding motif is not similar to a limb TF, and (E) randomized motifs of limb TFs.

(B,D,F). Equivalent tests considering the 575 CNEs diverged in subterranean mammals that overlap eye regulatory data.

Regardless of the motifs used to score the CNEs, snakes and subterranean mammals have no significant tendency to preserve binding sites, which shows that there is no selective loss of binding sites for limb or eye TFs but rather general sequence divergence. In contrast, sequence conservation in other species often results in a tendency to preserve predicted binding sites (matches to the TF motif), even if these binding sites occur purely by chance as for randomized TF motifs. As expected, the significances are substantially lower (compare to Figure 4A,C), suggesting that the respective predicted binding sites occur less often than predicted binding sites of limb/eye TFs.

As in Figure 4A/C, the difference in the median motif scores is shown on the x-axis. *p*-values were computed with a one-sided Wilcoxon rank sum test and corrected for multiple testing using the Benjamini & Hochberg method.

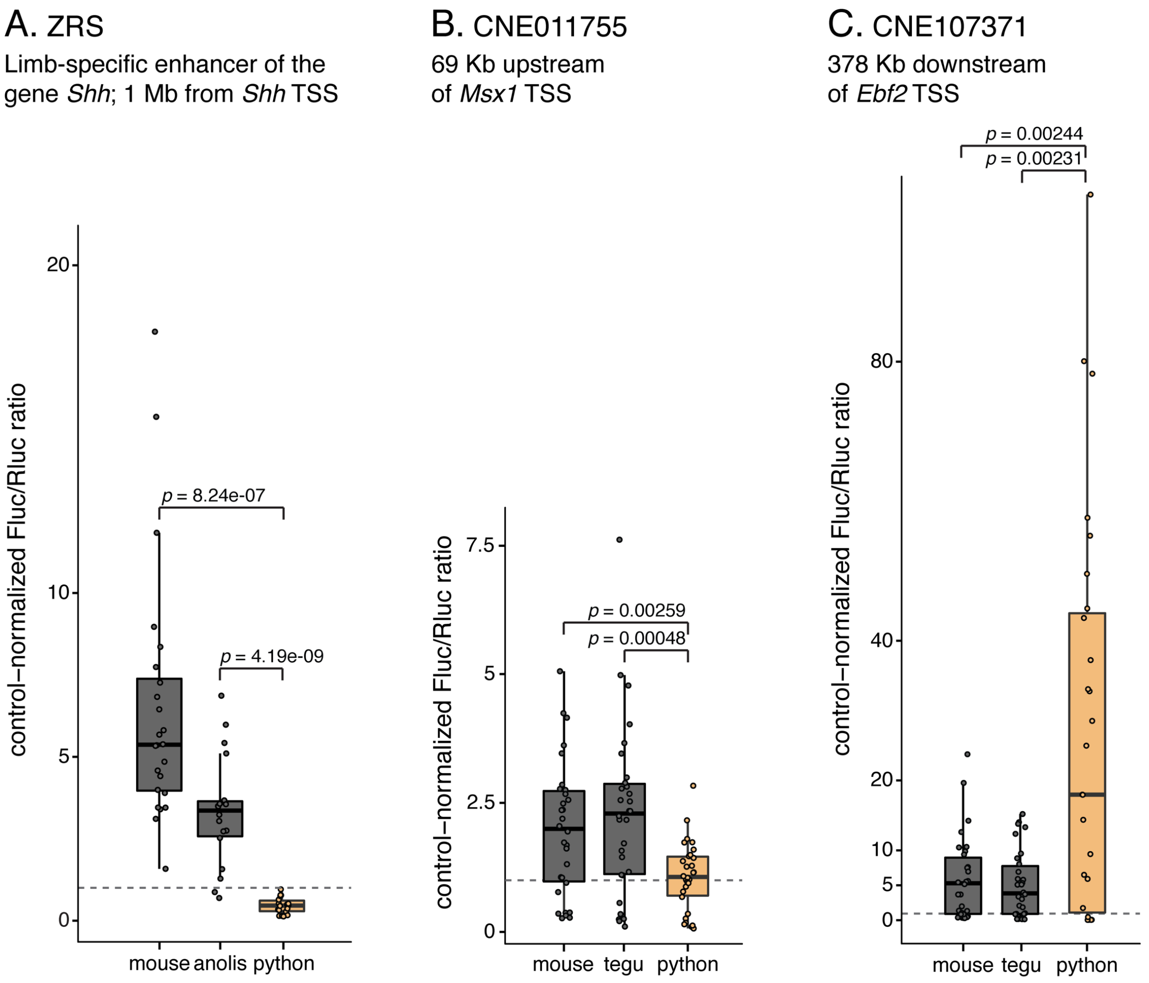

Supplementary Figure 15: Luciferase assays to compare regulatory activity of CNEs from limbed and limbless species.

(A) CNE overlapping the *Shh* ZRS limb enhancer. While the sequence of mouse and the green anole lizard has enhancer activity, the sequence of the python does not. This expression pattern recapitulates previously published results [16,17], thus serves as a positive control for the assay.

(B) CNE011755, which overlaps tegu lizard limb ATAC-seq, HOXA13 and HOXD13 ChIP-seq [11], H3K27ac marks [11,18], and a hindlimb enhancer detected from chromatin capture and histone modification marks [1]. This CNE is located 69 Kb upstream of *Msx1*, an important gene in the *Bmp* pathway [19]. Both the mouse and tegu lizard sequences have significantly higher enhancer activity compared to the python sequence, suggesting that sequence divergence resulted in loss of enhancer activity in the python.

(C) CNE107371, which overlaps tegu lizard limb ATAC-seq, mouse HOXA13 ChIP-seq [11], and a hindlimb enhancer detected from chromatin capture and histone modification marks [1]. This CNE is located 378 Kb downstream of gene *Ebf2*, potentially downstream effector of hedgehog signaling [20]. The python sequence drives significantly higher levels of expression compared to the mouse and tegu lizard

sequences, suggesting that sequence divergence in the python released a potential repressing activity. This is consistent with previous findings that repressing histone H3K27me3 marks overlap this locus in mouse [1].
p-values were computed with a two-sided Wilcoxon rank sum test.

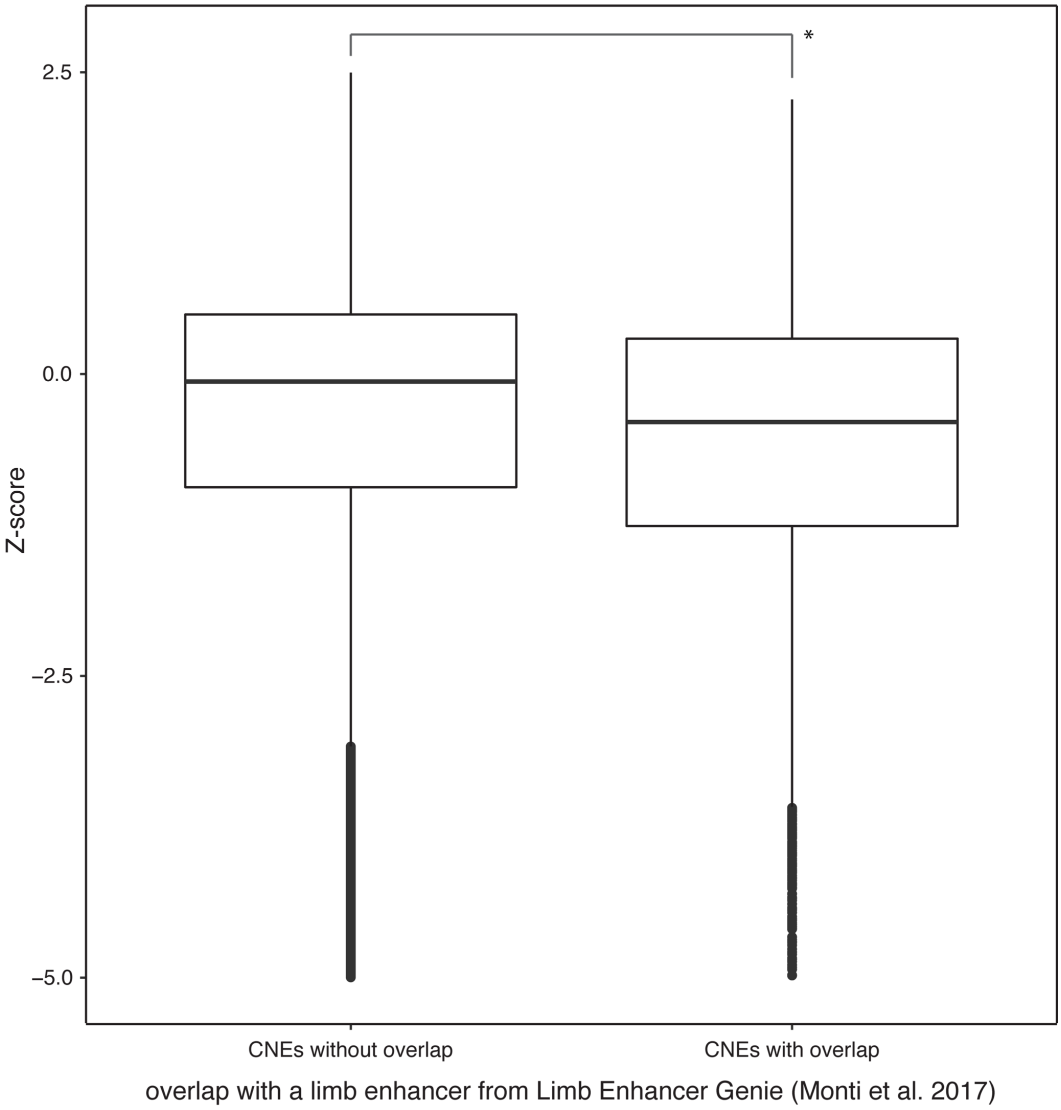

Supplementary Figure 16: Z-scores of the 5,786 CNEs that overlap a limb enhancer from Limb Enhancer Genie [21] are significantly lower than Z-scores of 135,824 CNEs that do not overlap these limb enhancers (two-sided Wilcoxon rank sum test $p < 2.2e-16$). For visual clarity, we cut the Y-axis at a Z-score of -5 and thus do not show outliers.

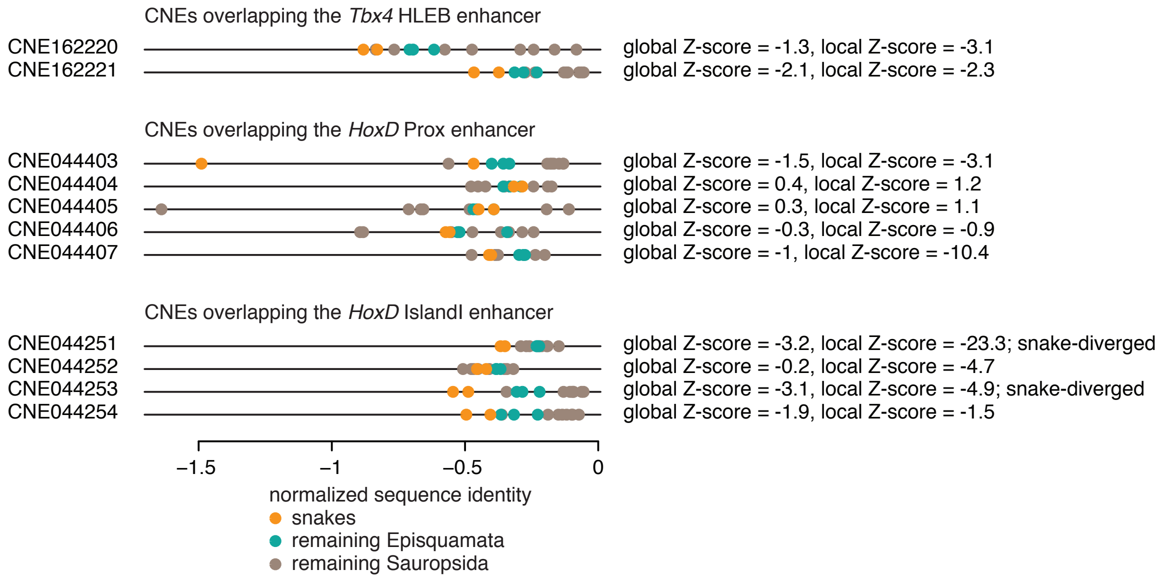

Supplementary Figure 17: Distribution of normalized sequence identity values for the CNEs that overlap known limb regulatory elements.
CNEs that overlap the HLEB enhancer regulating *Tbx4* (top) and the Prox enhancer regulating HoxD genes (middle) are not significantly diverged in snakes, even though some of these CNEs show some degree of sequence divergence. Two of the four CNEs that overlap the 2.7 Kb Island I, another enhancer regulating HoxD genes in the limb and genitals, are significantly diverged in snakes, with local and global Z-scores < -3 (CNE044251 and CNE044253; bottom).

Supplementary References

1 Andrey, G. *et al.* Characterization of hundreds of regulatory landscapes in developing limbs reveals two regimes of chromatin folding. *Genome Res* **27**, 223-233, doi:10.1101/gr.213066.116 (2017).

2 McLean, C. Y. *et al.* GREAT improves functional interpretation of cis-regulatory regions. *Nat Biotechnol* **28**, 495-501, doi:10.1038/nbt.1630 (2010).

3 Casper, J. *et al.* The UCSC Genome Browser database: 2018 update. *Nucleic Acids Res*, doi:10.1093/nar/gkx1020 (2017).

4 Birnbaum, R. Y. *et al.* Coding exons function as tissue-specific enhancers of nearby genes. *Genome Res* **22**, 1059-1068, doi:10.1101/gr.133546.111 (2012).

5 Visel, A., Minovitsky, S., Dubchak, I. & Pennacchio, L. A. VISTA Enhancer Browser--a database of tissue-specific human enhancers. *Nucleic Acids Res* **35**, D88-92, doi:10.1093/nar/gkl822 (2007).

6 Martinelli, D. C. & Fan, C. M. Gas1 extends the range of Hedgehog action by facilitating its signaling. *Genes Dev* **21**, 1231-1243, doi:10.1101/gad.1546307 (2007).

7 Cotney, J. *et al.* Chromatin state signatures associated with tissue-specific gene expression and enhancer activity in the embryonic limb. *Genome Res* **22**, 1069-1080, doi:10.1101/gr.129817.111 (2012).

8 Lachke, S. A. *et al.* Mutations in the RNA granule component TDRD7 cause cataract and glaucoma. *Science* **331**, 1571-1576, doi:10.1126/science.1195970 (2011).

9 Kleinjan, D. A. *et al.* Aniridia-associated translocations, DNase hypersensitivity, sequence comparison and transgenic analysis redefine the functional domain of PAX6. *Hum Mol Genet* **10**, 2049-2059 (2001).

10 Vokes, S. A., Ji, H., Wong, W. H. & McMahon, A. P. A genome-scale analysis of the cis-regulatory circuitry underlying sonic hedgehog-mediated patterning of the mammalian limb. *Genes Dev* **22**, 2651-2663, doi:10.1101/gad.1693008 (2008).

11 Sheth, R. *et al.* Distal Limb Patterning Requires Modulation of cis-Regulatory Activities by HOX13. *Cell Rep* **17**, 2913-2926, doi:10.1016/j.celrep.2016.11.039 (2016).

12 Infante, C. R., Park, S., Mihala, A. G., Kingsley, D. M. & Menke, D. B. Pitx1 broadly associates with limb enhancers and is enriched on hindlimb cis-regulatory elements. *Dev Biol* **374**, 234-244, doi:10.1016/j.ydbio.2012.11.017 (2013).

13 Corbo, J. C. *et al.* CRX ChIP-seq reveals the cis-regulatory architecture of mouse photoreceptors. *Genome Res* **20**, 1512-1525, doi:10.1101/gr.109405.110 (2010).

14 Hao, H. *et al.* Transcriptional regulation of rod photoreceptor homeostasis revealed by in vivo NRL targetome analysis. *PLoS Genet* **8**, e1002649, doi:10.1371/journal.pgen.1002649 (2012).

15 Samuel, A., Housset, M., Fant, B. & Lamonerie, T. Otx2 ChIP-seq reveals unique and redundant functions in the mature mouse retina. *PLoS One* **9**, e89110, doi:10.1371/journal.pone.0089110 (2014).

16 Leal, F. & Cohn, M. J. Loss and Re-emergence of Legs in Snakes by Modular Evolution of Sonic hedgehog and HOXD Enhancers. *Curr Biol* **26**, 2966-2973, doi:10.1016/j.cub.2016.09.020 (2016).

17 Kvon, E. Z. *et al.* Progressive Loss of Function in a Limb Enhancer during Snake Evolution. *Cell* **167**, 633-642 e611, doi:10.1016/j.cell.2016.09.028 (2016).

18 VanderMeer, J. E., Smith, R. P., Jones, S. L. & Ahituv, N. Genome-wide identification of signaling center enhancers in the developing limb. *Development* **141**, 4194-4198, doi:10.1242/dev.110965 (2014).

19 Lallemand, Y. *et al*. Analysis of Msx1; Msx2 double mutants reveals multiple roles for Msx genes in limb development. *Development* **132**, 3003-3014, doi:10.1242/dev.01877 (2005).
20 Dubois, L. & Vincent, A. The COE--Collier/Olf1/EBF--transcription factors: structural conservation and diversity of developmental functions. *Mech Dev* **108**, 3-12 (2001).
21 Monti, R. *et al*. Limb-Enhancer Genie: An accessible resource of accurate enhancer predictions in the developing limb. *PLoS Comput Biol* **13**, e1005720, doi:10.1371/journal.pcbi.1005720 (2017).
