# Supplementary material for: Phenotype loss is associated with widespread divergence of the gene regulatory landscape in evolution
Source: Nat Commun. 2018 Nov 9;9:4737. doi: 10.1038/s41467-018-07122-z (PMC6226452; doi:10.1038/s41467-018-07122-z)
Supplement: Supplementary file 3 — Description of Additional Supplementary Files [file 41467_2018_7122_MOESM3_ESM.pdf]

Supplementary Data legends

Data 1
Overview of the generated sequencing data
Data 2
Comparison of assembly completeness
Data 3
Species and assemblies used for the limb study
Data 4
Tegu lizard genomic coordinates and additional information of all 5,439 snake-diverged CNEs.
Data 5
Tegu lizard genomic coordinates of the regulatory domains of 439 genes involved in limb development.
Data 6
Tegu lizard genomic coordinates of the regulatory domains of 439 genes involved in limb development.
Data 7
Tegu lizard genomic coordinates of tegu limb-specific ATAC-seq peaks.
Data 8
Enrichment of snake-diverged CNEs with regulatory datasets.
Data 9
Mouse and Tegu lizard genomic coordinates of experimentally validated limb enhancers.
Data 10
Enrichment of snake-diverged CNEs with eye regulatory datasets.
Data 11
Species and assemblies used for the eye study
Data 12
Mouse mm10 genomic coordinates and additional information of all 9,364 CNEs diverged in the vision-impaired subterranean mammals.
Data 13
Mouse mm10 genomic promoter coordinates of 64 diverged eye-related genes.
Data 14
Enrichment of CNEs diverged in vision-impaired subterranean mammals with all MGI phenotypes.
Data 15
Enrichment of CNEs diverged in vision-impaired subterranean mammals with MGI phenotypes, after excluding the 64 diverged eye-related genes.
Data 16
Mouse mm10 genomic coordinates of eye-specific ATAC-seq peaks.
Data 17
Enrichment of CNEs diverged in vision-impaired subterranean mammals with regulatory datasets.
Data 18

Overlap between conserved and non-conserved limb regulatory elements with genital regulatory elements.

Data 19

Transcription factor motif clusters.

Data 20

List of limb-related transcription factors and their respective binding motifs.

Data 21

List of eye-related transcription factors and their respective binding motifs.

Data 22

CNE sequences that were tested in luciferase assays.

Data 23

List of softwares, versions, and corresponding parameters used for all analyses.
